# A tool for efficient and accurate segmentation of speech data: announcing POnSS

**DOI:** 10.3758/s13428-020-01449-6

**Published:** 2020-08-31

**Authors:** Joe Rodd, Caitlin Decuyper, Hans Rutger Bosker, Louis ten Bosch

**Affiliations:** 1 Max Planck Institute for Psycholinguistics, Radboud University, Postbus 310 6500AH, Nijmegen, The Netherlands; 2 Centre for Language Studies, Radboud University, PO Box 9103, 6500 HD, Nijmegen, The Netherlands

**Keywords:** Speech data, Segmentation

## Abstract

Despite advances in automatic speech recognition (ASR), human input is still essential for producing research-grade segmentations of speech data. Conventional approaches to manual segmentation are very labor-intensive. We introduce POnSS, a browser-based system that is specialized for the task of segmenting the onsets and offsets of words, which combines aspects of ASR with limited human input. In developing POnSS, we identified several sub-tasks of segmentation, and implemented each of these as separate interfaces for the annotators to interact with to streamline their task as much as possible. We evaluated segmentations made with POnSS against a baseline of segmentations of the same data made conventionally in Praat. We observed that POnSS achieved comparable reliability to segmentation using Praat, but required 23% less annotator time investment. Because of its greater efficiency without sacrificing reliability, POnSS represents a distinct methodological advance for the segmentation of speech data.

## Introduction

In many speech-based disciplines, the availability of adequately segmented and transcribed speech corpora is essential for designing and benchmarking computational models of speech processing and for sharpening theories of speech production and perception. Many of the speech databases available to date(e.g., via, The Language Archive, 2019; European Language Resources Association, 2019; Linguistic Data Consortium, 2019) have been (at least partly) enriched with a verbatim word-level and/or a phonetic transcription.

Speech transcription concerns the generation of a verbatim textual record of speech. The related process of segmentation concerns additionally determining when the transcribed words and segments occur in a speech recording. This article primarily addresses segmentation. Constructing transcriptions and segmentations typically involves three challenges. The first challenge is to take into account the purpose of the segmentation for determining the desired granularity level for the segmentation units. Due to fine phonetic details (Hawkins, 2003) and reduction phenomena (Ernestus & Warner, 2011), word-based transcriptions are much easier and faster to construct than high-quality finer-grained faithful phonetic segmentations. Rough, errorful transcription may be sufficient for text query-based services, and may be quickly constructed. Segmentation of varying degrees of accuracy may be required for rich diarization of meetings, or for the adaptation of acoustic models in automatic speech recognition (ASR). Language research represents a highly niche segmentation usage case with its own specific requirements and constraints.

The second challenge is the construction of the segmentation itself. This is not a trivial task. One may perform segmentation by hand or apply an automatic speech segmentation system, or a combination of these. Over the last decades, several tools have been developed to ease this task (see, e.g., van Bael et al.,, 2007; Lecouteux et al.,, 2012). In general, there is a clear trade-off between the invested time on the one hand and the quality of the resulting segmentation on the other (Rietveld et al., 2004).

The third challenge is the validation of the segmentation. Manual or automatic segmentations may be validated in terms of their resemblance to each other, or to another “expert-based” hand-crafted reference segmentation. Alternatively, they may be assessed by using e.g., the inter-rater or inter-system agreement as objective function. However, since symbolic segmentation cannot fully represent the subtle phonetic details in speech, the status of a “reference” segmentation as a single reference for the quality of other segmentations might be questionable a priori. In addition, the validation procedure will largely depend on the purpose. For example, verbatim “summary” transcriptions of meetings may be of sufficient quality to serve a service based on text queries, but still far from sufficient for the development or adaptation of acoustic models in ASR systems.

In this paper, we focus on the construction of segmentations at the word level, given a large collection of speech recordings. Several linguistic research tools are available for semi-manually segmenting, annotating, or labeling speech corpora. Tools may combine multiple functionalities such as speech recognition, speaker identification, and diarization to provide real-time and/or offline transcription of audio recorded in various conditions. Based on ASR approaches (e.g., Young et al.,, 2006; Povey et al., 2011), segmentation and transcription can be done automatically or semi-automatically. We will use the term “forced alignment” to refer to automatic segmentation of speech data using ASR where a transcription already exists, and the term “recognition” to refer to generation of a segmentation without a pre-existing transcription.

A number of pre-built, pre-trained forced alignment systems are available. The dominant systems are web(MAUS) (Schiel, 2015), FAVE/P2FA (Yuan & Liberman, 2008), and ProsodyLab-Aligner (Gorman et al., 2011), which are underlyingly based on the HTK ASR tooklit (Young et al., 2006) and MFA (McAuliffe et al., 2017), which is underlyingly based on the Kaldi ASR toolkit (Povey et al., 2011). HTK is based on a Gaussian mixture model—hidden Markov model acoustic models, while Kaldi is based on deep-learning networks instead of Gaussian mixtures.

Further systems aim to improve the usability of forced alignment, for instance by integration with Praat (the EasyAlign system, Goldman, 2011); focus on specific usage cases; or on less well-resourced languages. Braunschweiler et al., (2010) present a “lightly supervised forced aligner” where the forced alignment is done by using the transcriptions that are output from an automatic recognition step to be able to segment very long recordings, such as read speech gathered from audiobook recordings. (Stan et al., 2016) propose a system consisting of a Gaussian mixture model-based voice activity detector and a *grapheme*-based speech aligner, which they propose as particularly suitable for working with lesser-resourced languages. SPPAS is an alignment system that focuses on accurate detection of non-speech components of the signal, such as laughter and backchannel noises (Bigi and Meunier, 2018).

A potentially important facet of the reliability and robustness of forced alignment systems is how successfully acoustic models or features are adapted to the idiosyncrasies of individual speakers. This can be achieved by making use of i-vectors, maximum likelihood linear transform (MLLT), and linear discriminant analysis (LDA), possible in e.g., MFA (McAuliffe et al., 2017). Another important concern is the handling of out-of-vocabulary words. Words that appear in the system’s dictionary can be used in the transcription, but out-of-vocabulary words must first be processed by, e.g., grapheme-to-phoneme systems before they can be added to the aligner’s dictionary. In order to mitigate this out-of-vocabulary issue, the pronunciation dictionaries in PLA and FAVE were combined into one Arpabet-based dictionary, which was used across all three aligners for training (MFA, PLA) and alignment (MFA, PLA, FAVE).

Despite the variation in modeling techniques underlying these automatic forced alignment systems, and the various special-use systems, the quality of automatically generated segmentations still unavoidably depends on the acoustic quality of the recordings (presence of background noise, interference from speakers, cross-talk, echo etc.) and the degree of match between input speech signal and the speech material used for training the ASR (dialects, accents, age, speaking style, mood, etc.).

The recent advent of deep-learning techniques, together with improved computational power and availability of data, has lead to significant improvements in the performance of ASR systems. Despite these substantial improvements in their quality and practicality, fully automatic approaches to the segmentation of speech data for research purposes is still faced with challenging issues (Hannun et al., 2014), especially for under-represented languages (e.g., Bhati et al.,, 2019) and in case of more complex types of speech (pathological speech, multi-speaker recordings, recordings in adverse listening conditions, disfluent, highly reduced spontaneous speech). The aim of segmentation is often different in different research domains: the goals of the researcher in segmenting a speech dataset (precise information about the timing of features of speech) is somewhat (but increasingly) at odds with the big-data oriented requirements of modern commercial ASR research (Jurafsky & Martin, 2008; for zero-resourced languages there are alternatives, see e.g., Prasad et al.,, 2019). Furthermore, as long as completely automatic approaches are unable to deliver the reliability that researchers seek, human intervention will remain essential. A serious drawback of human intervention is its repetitive and time consuming character, putting it at risk of poor task execution, and therefore unreliable data.

The manual annotation of speech data is performed with specialized software. Several tools (e.g., the DART tool, Weisser, 2016) provide multiple interactive annotation functions, and allow special tools for those features that require post-processing. Praat (Boersma & Weenink, 2019) allows the user to manually segment and transcribe speech corpora using different tiers. EMU (Winkelmann et al., 2017) offers similar segmentation and transcribing possibilities as Praat, but in a web interface and in combination with a sophisticated database to store and manage speech data, segmentations and annotations. Despite the availability of these tools, the creation and checking of a segmented and transcribed speech corpus is still a considerable effort.

### This study

In this article, we discuss POnSS (Pipeline for Online Speech Segmentation), a system we have created and used for segmentation work for a number of recent studies involving large-scale segmentation (Rodd et al., 2019a, 2020, under review). With POnSS, we sought to improve the efficiency of the word segmentation task for human annotators. The aim of POnSS differs from, for instance, EMU (Winkelmann et al., 2017) in that we focus on optimizing a single task that takes a large amount of annotator time, rather than developing a fully featured speech data management system.

POnSS achieves its efficiency through combining forced alignment with manual checks and correction, an easy-to-use browser interface and, most innovatively, through subdividing the manual component of the overall task into subtasks and distributing them at the level of individual word recordings over annotators. To our knowledge, this task subdivision approach has not been tried before. In constructing POnSS, aside from segmenting our own datasets, our aim was to provide a practical implementation of a distributed, subdivided segmentation system, as well as to evaluate the reliability and efficiency of such an approach. We perform this evaluation in comparison to a conventional segmentation of the same data, performed using TextGrids in the phonetics software Praat (Boersma & Weenink, 2019), after forced-alignment bootstrapping.

The data that we use in the evaluation of POnSS come from Experiment 2 of the PiNCeR corpus (Rodd et al., 2019a). In that experiment, 13 speakers had to name pre-familiarized Dutch (C)CV.CVC words (e.g., *snavel* [snavel] “beak”) from line drawings displayed in groups of eight arranged on a “clock face”. A cursor moved clockwise from picture to picture to indicate at which of three trained rates (fast, medium, and slow) participants were required to name the pictures. Each trial of the experiment was recorded separately. The task was relatively difficult, meaning that speakers omitted or mispronounced words in many trials. On average, trials contained 6.39 correctly pronounced words that were ultimately analyzed, the modal number of included words was seven. Applying POnSS to the PiNCeR data provides a test case where the words to be produced were known in advance, but not reliably present, a particularly difficult case for forced alignment. This is in contrast to data where it is not reliably known what will be said. POnSS can be useful for this latter type as well, but with a few adjustments, as explained below.

## POnSS

**Fig. 1:**
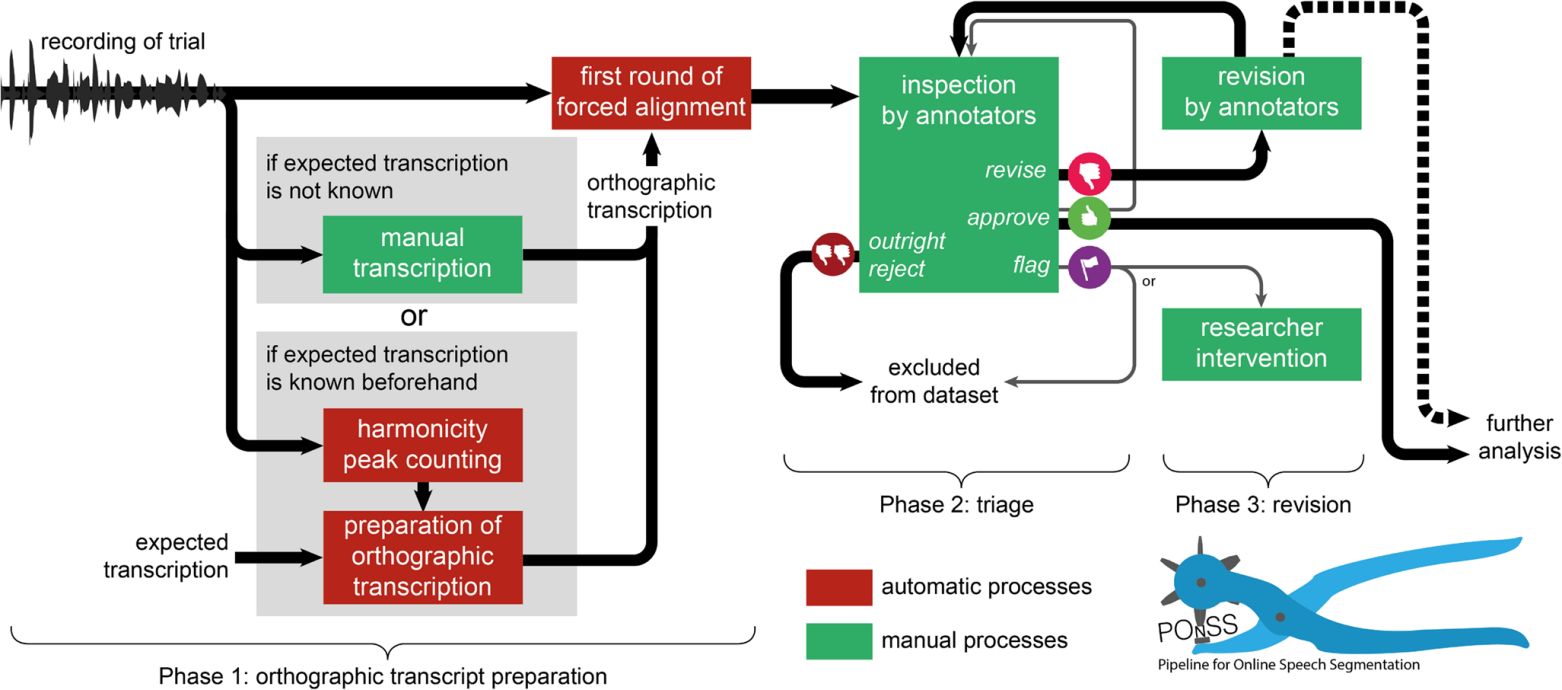
A diagrammatic representation of the annotation process. See the text for full details

POnSS is a multi-step acoustic analysis and forced alignment pipeline to segment speech materials, intended to be used by a panel of phonetically trained annotators, with each annotator seeing a partially overlapping part of the dataset. This pipeline is illustrated diagrammatically in Fig. 1. POnSS divides the work of speech segmentation into three broad phases; orthographic transcript preparation, triage, and retrimming, each stage combining both manual and automatic processes. The manual processes are standalone, and each unit of work is small, meaning that annotators can themselves choose which of the tasks they do, and for how long, as long as there are materials available to be worked on.

### Phase 1: orthographic transcript preparation

The first phase of POnSS is the preparation of an orthographic transcription. POnSS includes both a manual procedure for when the exact word sequence is not known, and a fully automatic procedure for when an expected trial transcription is known beforehand.

#### Manual transcription

For datasets and experiments where speakers may be particularly errorful in their speech, or where no specific expected transcription exists, POnSS includes a module that facilitates the full manual word-level transcription of the speech data. This approach was used to transcribe the data for (Rodd et al., under review), where the vocabulary of possible words was known, but we expected the speakers to make many errors given the longer trials. We expected these frequent errors to make a transcription based on the picture sequence insufficiently reliable for forced alignment. First, silence/pause detection divides the trial recordings into audio chunks with a duration of minimally 5 s and maximally 30 s. These chunks are inserted into the database.

**Fig. 2:**
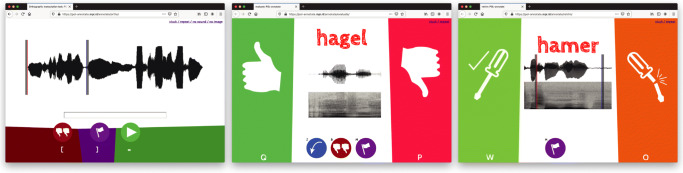
Screenshots of the browser interfaces for the orthographic transcription (*left*), triage (*middle*), and retrimming tasks (*right*) in POnSS

Annotators use a browser interface (Fig. 2, left panel) to transcribe each chunk individually, orthographically. Annotators are asked to use real (canonical) word forms, also in the cases where speakers use reduced pronunciation variants. When the experiment involves a constrained vocabulary of words that can appear, the interface is able to suggest word completions as annotators type, which reduces the number of required keystrokes.

#### Harmonicity-aided automatic procedure

For datasets where the expected ordering of words is known, POnSS offers a fully automatic transcription generation procedure. This begins with the analysis of the harmonicity (autocorrelation method, default settings) of the trial recordings, using Praat (Boersma & Weenink, 2019). In the analysis of the PiNCeR dataset, each harmonicity peak is assumed to correspond to one vowel in the recording, allowing the number of disyllabic words actually produced to be estimated. This may serve as a check for the degree of match between audio and prompted text. In the PiNCeR dataset, in which speakers were asked to pronounce eight disyllabic words in a sequence at varying speaking rates, we observed that when speakers produced fewer than the full eight words, words occurring later in the sequence were much more frequently omitted than earlier ones. Based on this observation, the peak counts were used to produce candidate orthographic transcriptions for use in the forced alignment procedure. In the case of the PiNCeR data, if 15 or 16 harmonicity peaks were detected (indicating equally many syllables), all eight words were included in the transcription. If 13 or 14 peaks were detected, the first seven words were included, and so on. This is done with the aim of achieving better forced alignment results than simply forced aligning against the orthographic transcription including all eight words would do.

#### Forced alignment

Once an orthographic transcription is available of a trial or chunk, forced alignment is performed using the application programming interface (API) to webMAUS (Schiel, 2015), which offers good-quality forced alignment for Dutch and other languages using HTK (Young et al., 2006). The resulting word onsets and offsets are used to cut out the audio chunks related to individual words from the longer trial audio recording. We term the resulting labeled chunks of audio “word candidates”, since we cannot yet be sure of the accuracy of the segmentation.

### Phase 2: triage

In the triage phase, annotators use a browser interface in which each word candidate is presented individually, displaying the transcription, waveform, and spectrogram. The spectrogram and waveform include a “shoulder” of adjacent material either side of the word candidate, made translucent in the waveform (see Fig. 2, middle panel). The audio plays immediately on loading, and can be replayed as often as required by pressing the tab key or clicking on the waveform. Words are selected randomly from the stack of word candidates that still need to be triaged. The annotator’s task is to choose from one of four options:

1. Mark the word candidate as correctly annotated; our annotators were instructed to decide whether the “complete word is isolated, with no extraneous material included”. (thumbs up in middle panel of Fig. 2)
2. Mark the word candidate as requiring further attention in the retrimming phase. (thumbs down)
3. Discard the word candidate because it contains non-speech, for instance environmental noise or a cough. (double thumbs down)
4. Mark the word candidate as requiring manual intervention, for instance because a speech error (such as a mispronunciation or naming a different word) was made. In our case, these words were also excluded, but POnSS can collect them for later intervention by the researcher. (flag)

Each of these options is associated with a button in the browser interface and associated with a specific key. As soon as a decision is made, the interface automatically proceeds to the next word candidate.

Depending on decisions made by the researchers, word candidates that are marked as good are either returned to the “stack” to be checked again until the word candidate has been approved by a defined quorum of annotators, or removed from the stack and enter the dataset. In our case, we set a target that 20% of the word candidates should be triaged more than once. Which word candidates that passed the triage were revisited was decided randomly.

### Phase 3: retrimming

In the retrimming phase, the onset and offset boundaries of the fraction of word candidates that were marked by annotators as requiring retrimming are adjusted. Again, a browser interface was used (Fig. 2, right panel). The label, spectrogram and waveform of each word candidate are again presented on screen. This time, the annotator drags the onset and offset boundaries with the mouse to correct the segmentation. They have three options:

1. Report that they successfully corrected the segmentation (screwdriver with check mark in right panel of Fig. 2)
2. Request that the word candidate should return, with more margin (snapped screwdriver)
3. Mark the word candidate as requiring manual intervention, for instance because a speech error was made. In our case, these words were also excluded, but POnSS can collect them for later intervention by the researcher (flag)

Depending on researcher-controlled settings, word candidates that annotators report as successfully corrected can be returned to the triage “stack” to be double checked, or they can be removed from the stack and enter the dataset.

### Computational implementation

Most components of POnSS are implemented in Python as a web application using the Django framework (Holovaty & Kaplan-Moss, 2009). The interfaces themselves are implemented using HTML, CSS, and JavaScript. In-progress segmentation data, along with all meta-data about the annotators’ interaction with the system are stored in a PostgreSQL database.

Although POnSS at present has its own PostgreSQL back-end, elements of the pipeline and the orthographic transcription, triaging and retrimming task interfaces could be relatively easily coupled to another speech data management system, such as EMU-SDMS (Winkelmann et al., 2017).

All code implementing POnSS is available at https://git.io/Jexj3, along with the Supplementary Materials.

## Baseline manual segmentation method

We designed a baseline task that is typical for the type of segmentation projects that are conducted for production data in psycholinguistics (for instance, Zormpa et al., 2019; Sjerps et al., 2019), combining forced-alignment and Praat TextGrid annotation.

We selected a sample of 468 trial recordings from Experiment 2 of the PiNCeR corpus (Rodd et al., 2019a) that were balanced for speaking rate and speaker. These trial recordings were forced-aligned using webMAUS (Schiel, 2015), based on the expected word productions, and Praat TextGrids were prepared with the forced-alignment result. A panel of seven trained annotators, all of whom were native speakers of Dutch, were asked to correct the MAUS transcriptions of all of the trials in the sample in Praat. They were employed as research assistants and worked on this project as part of their paid work. In contrast to typical practice, where only one annotator looks at each recording, in this case, all seven annotators looked at all 468 trials. A script in Praat selected an audio file and the corresponding preprocessed TextGrid and opened both. Annotators were asked to check the boundaries for word onset and offset and move them if necessary, and check the labeling of the words. Annotators clicked on a “continue” button to save the adjusted TextGrid and load data for the next trial.

## Assessing the reliability of transcription data

Like all human-derived data generation processes, speech segmentation/annotation procedures are liable to various kinds of unreliability. Although one intuitively understands what it means for data to be reliable, formalizing this into a working definition is less straightforward. A frequent definition is that reliability is “the consistency with which a measure assesses a given trait” (e.g., Bartko & Carpenter, 1976), framing reliability as synonymous with reproducibility. In the domain of speech segmentation, this definition implies we should be assessing how consistent annotators are in the boundary time stamps that they assign. This could be operationalized within annotators working on the same dataset multiple times (as a kind of test-retest reliability) or between annotators (as a kind of inter-rater reliability).

Relatively little attention has been given to the concept of reliability in the temporal dimension of speech data annotation, with discussion of (un)reliability primarily focused on the label dimension (e.g., Gut & Bayerl, 2004; Widlöcher & Mathet, 2012; Mathet & Widlöcher 2011, 2015; Yoon et al., 2004).

Outside the speech domain, a number of inter-rater reliability coefficients are prevalent (Popping, 1988). Many such coefficients are constructed with the assumption of categorically distinct data, assume precisely two raters, or assume that all raters will look at each case. A few coefficients are proposed as being suitable for continuous data, notably intraclass correlation (ICC; Bartko, 1966) and Krippendorff’s alpha (Krippendorff, 1970; Hayes & Krippendorff, 2007). Krippendorff’s alpha is broadly applicable to data of different forms, suitable for an arbitrary number of annotators, and tolerant of missing data. An alpha value of 1 indicates perfect reliability, an alpha value of 0 indicates the absence of reliability. Negative alpha values indicate above-chance systematic disagreement. In practice, standardized reliability coefficients have not gained traction in speech research, and it is typical to calculate the percentage of segmentations that fall within some tolerance relative to another annotator’s segmentation, or relative to a gold standard segmentation, which may be hard to motivate (Ernestus et al., 2015; Raymond et al., 2002; Kipp et al., 1997).

Because of its broad applicability and comparability, we initially selected Krippendorff’s alpha as the metric to be used to evaluate POnSS. We intended to use bootstrap re-sampling to create variance in the coefficient, to allow statistical comparison across samples annotated by the baseline method and by POnSS. However, we found disturbingly little variation in the alpha coefficients that we calculated. To explore this systematically, we set about exploring the properties of Krippendorff’s alpha, ICC and “percentage within tolerance” measures in the context of the baseline annotation data. We did this by adding or removing noise to the individual segmented onset and offset times in the dataset of word segmentations performed with the baseline method, and calculating the coefficients for each “tweaked” dataset. None of the tested coefficients were able to distinguish between datasets that we had artificially made more or less reliable, with Krippendorff’s alpha and ICC essentially exhibiting no variation. These simulations are reported in the Supplementary Materials.

### Distribution fitting approach

**Fig. 3:**
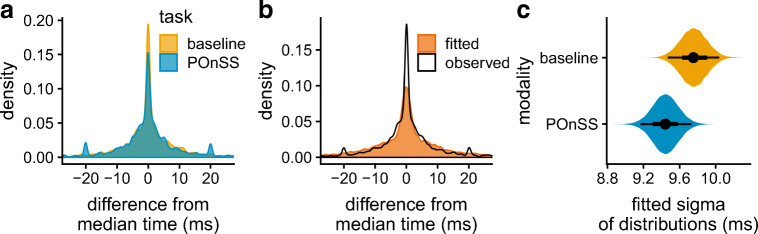
Panel **a**: the observed distributions of the difference between segmented times and the median segmentation for each word, for POnSS and manual annotation modalities (*colors*). Panel **b**: an example of the optimized mixture-model fit (*orange*) to the observed distribution of one of the samples (*black line*). Panel **c**: *Solid violins* show the posteriors of Model 1 (see text) for the effect of modality on the sigma, with median (*points*), 95% HDIs (highest density intervals, *thin black lines*) and 66% HDIs (*thick black lines*)

Given our conclusion that none of the established reliability metrics offered a sufficiently sensitive way to assess the reliability of our speech segmentation data, we developed an alternative approach based on distribution-fitting. This approach aims to quantify variability by finding the parameters of a model of the data-generating process that explains the variability in the word boundaries resulting from the segmentation process, rather than deriving a result directly from the outcomes. We consider the distribution of the differences between individual segmented onset and offset times and the median of all onset or offset times recorded for that same word across annotators. This distribution, illustrated in Fig. 3a, has both a high, narrow peak, and broad tails, and is centered around 0 ms, where there is no difference between an individual segmentation and the median of segmentations of the same material, which led us to fit it as a mixture of overlapping Gaussian distributions.

The model that we fit consists of a mixture of three Gaussian distributions. A Gaussian distribution is defined by two parameters, a central tendency (or mean, *μ*) and a standard deviation (*σ*). The *μ* and *σ* of each Gaussian distribution are therefore the parameters that we vary to find the best fitting mixture of Gaussians. The narrowest Gaussian captures the very best segmentations, where all annotators were in full agreement. This is constrained to *σ* values between 0.0001 and 2 ms. The second Gaussian captures segmentations that deviated somewhat from the median, constrained to *σ* values between 1.5 and 8 ms. The third Gaussian captures very poor segmentations, where boundaries were placed a long way from the median, constrained to *σ* values between 2.5 and 40 ms. The search regions overlap to keep the fitting as data-driven as possible. All three Gaussians have their *μ* parameter clamped at 0. The relative contribution of each Gaussian to the overall mixture is also parametrized, *θ*_*i*_ is the proportion of the mixture that is contributed by Gaussian *i*. Within a mixture, the *θ* s must sum to 1.0. In line with typical mixture-modeling practice, we refer to each of these Gaussians as a “component” of the overall distribution, there are thus three components, which we term “narrow”, “medium” and “wide”.

Once the mixture model has been fitted to the data, the resulting *σ* s, weighted by the *θ* s, quantify the reliability of the sampled segmentations. These could either be summarized as a weighted sum, or used for inference in a weighted regression, as we do in the next section.

## Analysis 1: Reliability of modalities

### Materials

To construct a dataset to evaluate the performance of POnSS, we combined the data from the baseline manual segmentations described in “Baseline manual segmentation method” and a subset of the word segmentations produced using POnSS for Experiment 2 of the PiNCeR corpus (Rodd et al., 2019a), namely word candidates that had been retrimmed minimally twice (as part of random double work to facilitate this investigation). As far as possible, the same words were used as in the baseline manual task. The panel of eight paid research assistant annotators who contributed to the POnSS data sample were similar in training and background to those who annotated for the baseline task, and included some of the same research assistants.

For each individual word token, the median word onset time across all annotators and both modalities (POnSS or baseline) was calculated. The same was done for the offset times. For each segmentation, the difference between the segmented onset and offset times and the medians was calculated. A balanced sample was taken for statistical modeling, including 300 onset segmentations and 300 offset segmentations for each modality for each of the speaking rates in the experimental data (fast, medium, slow). This sample is shown in Fig. 3a. In the distribution for POnSS (blue), there were small peaks at - 20 ms and + 20 ms. These likely emerged because it was possible to adjust the position of the boundaries during retrimming with the keyboard; pressing shift+left or shift+right moved the boundary 20 ms.

### Quantifying differences in reliability

To be able to identify the effects of modality and speaking rate on the fitted sigmas, we prepared a dataset that would allow us to predict the sigmas fitted in the Gaussian mixture model by modality and speaking rate. We constructed subsets of the test dataset that varied in the proportion to which each speaking rate or modality was represented. The proportions were predefined, at approximately 10% to approximately 80%, in steps of 10% for the rate conditions. For the modality conditions, we set the proportions of manual annotation to between 20% and 80%, again in steps of 10%. The different levels were exhaustively combined, meaning that 252 samples were constructed, for instance a sample might contain segmentations that were 30% POnSS segmentations and 70% baseline, 40% from the slow condition, 40% medium and 20% fast; a second sample might be 60% POnSS, 40% baseline, 50% slow, 10% medium, and 40% fast.

Next, we performed optimization to find, for each sample, plausible values for the parameters of the mixture model described in “Distribution fitting approach”. The *σ* of each Gaussian was a free parameter, as were the mixing proportions of the Gaussians (*θ*). The central tendency (*μ*) was always 0. The quality of the fit was quantified as the mean Kullback–Leibler divergence (KL) between the observed and the fitted distribution, and between the fitted distribution and the observed distribution. Optimization was performed using the hydroPSO implementation of the particle swarm algorithm in R (Zambrano-Bigiarini & Rojas, 2018), which performs efficient optimization by simulating a swarm of “particles” that attempt different parameter values. Sixty particles were simulated for maximally 2000 iterations. The parameter values (*θ* and *σ* s) of all 60 particles in the final iteration of the optimization were recorded, along with the achieved KL for that set of parameter values. In general, good fits are achieved of the fitted distribution to the observed distribution. A sample fit is shown in Fig. 3b.

### Inferential model

We then fitted a Bayesian regression model to quantify the influence of using POnSS in place of the baseline task. This model, and all further statistical models reported, were fitted with the R package brms (Bürkner, 2018), allowing us to fit Bayesian mixed-effects models in which the width of the fitted distributions is parameterized. Rather than dealing with binary decisions between significant and not significant, Bayesian regression focuses on quantifying uncertainty about the magnitude of an effect (e.g., Vasishth et al.,, 2018), so no *p* values are reported. Instead, we report the size of the effects we identify, in their relevant units, and where appropriate, standardized for comparability (Cohen’s *d*). All intervals reported are 95% highest density intervals (HDIs).

The model predicted the optimal sigmas found by the particle swarm optimization for each subset, on the basis of the proportion of each modality and each speaking rate, which varied between the subsets, as described in “Quantifying differences in reliability”. The interaction between modality and speaking rate was also included. We will refer to this model as Model 1. The model was sampled with the NUTS sampler with six chains of 4000 warm-up and 4000 test iterations. The model converged for all parameters, as assessed by the Gelman–Rubin diagnostic *R̂* being within 0.001 of 1.0.

Predictors were included for the proportion of POnSS segmentations, the proportion of segmentations of words from the fast condition and the proportion of segmentations of words from the slow condition. It was not necessary to include the proportion of manual annotations or the proportion of segmentations of words from the medium condition, since these are entirely correlated with the proportion of POnSS segmentations and the sum of the proportion of fast and slow, respectively. This is intuitively comparable to treatment coding of a categorical variable. For each of these linear predictors, a weakly informative prior was specified (*μ* = 0, *σ* = 5). A deviation-coded categorical predictor was included for component (narrow, medium, or wide), as were interactions between the categorical and linear predictors. The model fitted a Student’s *t* distribution, the *σ* and *ν* parameters, which were predicted by the component. Regression weights were applied, consisting of the fitted *θ* values associated with the relevant component, multiplied by 1 − the KL score achieved by the fitting. This means that the sigmas of the three mixture components contributed to the main effects in proportion to their weighting, and that the best fits contributed more than worse performing fits. Full details about the model are available in the Supplementary Materials.

No reliable difference emerged between POnSS and manual segmentations on medium-rate speech: - 0.31 ms [- 0.71, 0.084], though the central tendency suggests that, had only POnSS segmentations been present in a sample, we would expect to see marginally narrower distributions than in a sample annotated only by the manual method. This effect is depicted in Fig. 3c. This effect was involved in interactions, such that, with POnSS, reliability was marginally worse in the narrow component: 0.43 ms [0.14, 0.73], in the medium and wide components, the interaction effect was not distinct (medium: - 0.0052 ms [- 0.34, 0.34]; wide: - 0.43 ms [-1.1, 0.2]). A figure depicting these interactions is available in the Supplementary Materials (Figure S9). Had a sample only contained fast speech, we would expect wider distributions: 2.5 ms [2.1, 2.9]. No reliable difference emerged between medium and slow speaking rates: 0.051 ms [- 0.36, 0.46]. There were no reliable interactions between modality and rate (POnSS and fast rate: 0.12 ms [- 0.38, 0.63]; POnSS and slow rate: - 0.33 ms [- 0.79, 0.14]), suggesting that POnSS is equally reliable across speech that may be assumed to differ in style.

Together, these results indicate that segmentations performed with POnSS are at worst equally reliable as segmentations performed conventionally using Praat, and potentially slightly better.

## Analysis 2: Efficiency of modalities

To assess the efficiency of POnSS, we calculated how many annotator-hours would be required to yield 5000 correctly segmented words, using either POnSS or the baseline modality. The Praat script that was used in the baseline modality recorded the time when each trial recording was opened and saved. From these timestamps, we calculated the time spent on each trial, and divided that by the number of words that the annotator segmented in that trial. This yields the duration that was spent annotating each correctly segmented word.

Because words are not worked with sequentially in POnSS, establishing how much time was spent working on each individual word is less straightforward. The POnSS system records timestamps for the time that each word-candidate was presented to the annotator for triage, and the time when they finished interacting with it. The same was done for the retrimming task. Because POnSS allows each word in the dataset to be triaged and/or retrimmed by multiple different annotators, and some words that were ultimately rejected were also retrimmed, simply summing these times for the words in the finished dataset would underestimate how much time was spent working with a word candidate to result in a well segmented word in the finished dataset. In the baseline modality, this correction for missing words is already implicitly made, since the time spent working on a trial is divided by the number of resulting good words. Instead, we identified all interactions with each original word recording across both tasks, and summed all the time spent working with that word across annotators and tasks. We also checked whether the word was accepted into the finished dataset or not.

To be able to statistically model the time spent per successful word in each task, we adopted an approach akin to bootstrap resampling. For the baseline modality, we randomly selected 1000 samples containing 5000 words, and summed the time spent for each word together, to yield a distribution of time investments to segment a sample of 5000 words. For POnSS, for each sample, we started with a sample of 5000 words (that were either included or rejected from the finished database), and iteratively added more words until the sample contained 5000 non-rejected words. Around 5% of word candidates were rejected during triage or after retrimming, meaning they did not make it into the finished dataset. Again, we added the time spent for all (included and rejected) words in the sample together, yielding a distribution of time investments to segment 5000 included words.

**Fig. 4:**
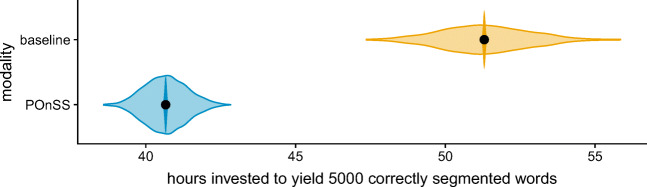
Distributions of bootstrap-resampled estimates of how many annotator hours it would take to yield 5000 well-segmented words by the two modalities (*translucent violins*). Overlaid are *solid violins* showing the posteriors of Model 2 for the effect of modality, with median (*points*), 95% and 66% HDIs are too narrow to see in the figure

The distribution of the time it took to yield 5000 correctly segmented words by each modality is shown in the translucent distributions in Fig. 4. Note that in this dataset, no manual transcription was required, since we used the harmonicity-aided automatic transcription generation procedure; in the baseline case, MAUS was used, meaning that the analogous part of the task was not used there either.

We fitted these distributions with a Bayesian regression model (Model 2). Like Model 1, Model 2 was sampled with the NUTS sampler with six chains of 4000 warm-up and 4000 test iterations. The model converged for all parameters, as assessed by the Gelman–Rubin diagnostic *R̂* being within 0.001 of 1.0. The model predicted the hours invested to yield 5000 correctly segmented words, with a deviation-coded categorical predictor for the modality (1 indicated the baseline method, -1 the POnSS method). A weakly informative prior was set for this predictor, a normal distribution centered at 0 with a *σ* of 5.44 h, meaning our expected effect size was 0, with a standard deviation of 1 Cohen’s *d*. For the model intercept, the prior was a Student’s-*t* distribution centered at 45.98, the average of all the data with a *σ* of 54.42 h and a *ν* (degrees of freedom) of 16.33, which are derived by scaling the recommended properties of this prior in brms to this dataset.

The posterior distributions of the model coefficients of interest are shown in Fig. 4, as solid violins. The difference between the two approaches in the time taken to yield 5000 correctly segmented words was very clear (difference between means: 11 h [11, 11], Cohen’s *d* = 2), such that segmentation using POnSS required 23% less investment of annotator time than the baseline method.

## Discussion

In this article we introduced POnSS, an online pipeline for the segmentation of speech data. POnSS is optimized for this single task, sacrificing functional flexibility in favor of time/effort efficiency.

We argued that, while fully automatic speech transcription and segmentation is gaining traction, for many purposes human intervention remains essential to ensure data quality in conditions adverse to speech segmentation. A key diagnostic for the quality of a speech segmentation is its reliability, conventionally defined in terms of reproducibility. We explored how two widely employed approaches to measuring reproducibility were sensitive to the kind of variance expected in speech segmentation data. From this analysis, we concluded that neither Krippendorff’s alpha nor simple percentage agreement within a tolerance were ideal ways to assess reliability in speech segmentation data, since they were not sensitive to artificial noise. In their stead, we proposed a reliability-quantification approach based on modeling the underlying error process as a mixture of Gaussian distributions, where the sigmas of the distributions quantify the reliability of the segmentation process.

We then turned to quantifying the consequences of segmenting to the word level with POnSS rather than with a conventional procedure using TextGrids in Praat preceded by naive forced-alignment. We analyzed the relative reliability and efficiency of POnSS. These analyses revealed that segmentation with POnSS was approximately equally reliable compared to conventional manual segmentation, and considerably faster. In the reliability analysis, we found that the sigmas fitted to the data segmented by POnSS were comparable to the sigmas fitted to the data segmented conventionally. The efficiency analysis showed that 23% less investment of annotator time was required to yield the same number of acceptable word transcriptions. In the efficiency analysis, the way that we compared the modalities slightly biases against POnSS, since we calculate the time investment based on our practice whereby some word-candidates got triaged and retrimmed multiple times by different annotators, while we assume that under the baseline modality, each word-candidate will only be worked on once. These findings license the further use of POnSS for segmentation of speech corpora. For the evaluation conducted here, we used data from the PiNCeR corpus (Rodd et al., 2019a). The PiNCeR corpus was a good test case, since it contains experimentally elicited, errorful speech, which is particularly challenging for forced alignment.

Aside from reliability and efficiency, a subordinate aim in developing POnSS was to improve the experience of the annotators, who consider segmentation to be the least preferred of the tasks that they perform as research assistants. Anecdotally, the annotators report POnSS to be preferable to work with, compared to conventional segmentation using TextGrids in Praat. This preference may be explained by a number of differences between POnSS and using TextGrids in Praat. Firstly, the knowledge that POnSS was designed with the goal of greater efficiency may in itself lead annotators to prefer POnSS. That the system is more ergonomic, requiring fewer keystrokes and mouse clicks, and less mouse movement because the interface is logically placed may also have contributed to this. Secondly, annotators may experience conventional segmentation as constraining, as they have to ensure that the work that they do is consistent with rigid protocols. Third, the choice of which subtask to perform may give annotators enough agency in POnSS to feel more in control of their own work. Furthermore, the annotators and project manager are freed from a number of meta-tasks inherent to conventional segmentation projects. These include the necessity to keep track of how far through a project they are and recording this to prevent double work; planning how many trials can be done in the time remaining until the next task begins; and ensuring that work is saved and archived. Finally, annotators may prefer POnSS due to its relatively colorful, appealing visual appearance. Future analysis might examine whether they work longer effective stints with POnSS than with the baseline task. In POnSS it is possible to employ aspects of “gamification”, for instance tracking and displaying each individual annotator’s longest streak of triage decisions or retrimmings performed within some time limit to boost motivation, though whether this would come at the cost of reliability would need to be established.

In POnSS, different component tasks of the overall segmentation project are separated out into small, easily explained and understood sub-tasks. This implies that the less taxing triaging task could potentially be adequately performed by entirely untrained annotators, through online crowd-sourcing systems such as Amazon’s Mechanical Turk (Buhrmester et al., 2018), or allowing paid members of an institute’s participant pool to segment data at home at their convenience. This would drastically reduce the wait for the researcher for completed segmentations, and free up trained research assistants for more productive and motivating tasks. Further careful pretesting is required to establish whether crowd-sourced, non-expert triage decisions are of equal quality to expert triage decisions, and to introduce data-quality controls like catch trials with known good answers.

Our aim with POnSS was to provide a practical implementation of a distributed, subdivided segmentation system, to be able to evaluate the efficiency and reliability of such an approach. As such, there are various researcher degrees of freedom, such as the length of chunks in the transcription task and the proportion of word candidates that are triaged and retrimmed multiple times that could influence the reliability and accuracy of the resulting segmentations. Optimal settings for these researcher degrees of freedom need to be explored more fully with various annotator populations and speech data types, which may allow further improvement on the efficiency benefit relative to Praat TextGrids reported here.

The test dataset that we used to evaluate POnSS was segmented to the word level, and we used other techniques to perform sub-word level analyses (Rodd et al., 2019b). The subtasks of POnSS are readily applicable to segmentation to other levels of granularity, too, and presumably would result in similar efficiency gains relative to conventional segmentation to the same level of granularity.

Intuitively, there is a hierarchy of difficulty of manual segmentation to different levels of granularity, related to the extent to which the units in question are identifiable from acoustic, waveform and spectrogram features. For this reason, segmentation to the utterance level is trivial, segmentation to the word level is fairly easy, segmentation to the syllable level somewhat more challenging, and segmentation to the phone level is highly challenging. This variable task complexity influences the maximally achievable reliability of manual segmentation. It seems unlikely that the properties of phone and syllable-level segmentation that make these tasks challenging in conventional segmentation would be affected much by using POnSS instead, so we assume that POnSS would offer similar advantages over conventional segmentation below the word level too, though further testing is required to be sure.

POnSS also includes a manual transcription component that makes the segmentation of spontaneous speech viable. Read speech resembling, for instance, TIMIT (Garofolo et al., 1993), which forms the basis of the training data for many ASR systems, may be forced aligned well enough to require only minimal checking of a sample to assess the suitability of the segmentation. POnSS could be trivially adapted to manage this forced alignment and perform this checking, bringing the productivity benefits of the POnSS subtasks and database system to this type of project as well, and potentially also the possibility of crowd-sourcing this work, as discussed previously. A further potentially highly productive extension to POnSS would be to apply its principles of task subdivision and its browser-based database system to annotations of multimodal datasets (e.g., audiovisual recordings). This would potentially speed up the annotation and coding of gesture and sign language materials.

The test dataset that we used was rather small in comparison to the kinds of segmentation projects performed for large scale research corpora and ASR training set development. For instance, the Spoken Dutch Corpus (Oostdijk, 2000), contains 116 times as many phonetically transcribed words, and the pre-trained English model for the Montreal Forced Aligner (McAuliffe et al., 2017) is trained on the LibriSpeech corpus (Panayotov et al., 2015), which is roughly 640 times larger. The very large size of this type of project highlights the relevance of the efficiency improvement realized by segmenting using POnSS.

In conclusion, POnSS offers reliable segmentation of speech materials to the word level, in an appealing form that makes efficient use of human input by combining human decisions with forced alignment.
